# Development of a Microfluidic Array to Study Drug Response in Breast Cancer

**DOI:** 10.3390/molecules24234385

**Published:** 2019-11-30

**Authors:** María Virumbrales-Muñoz, Megan K. Livingston, Mehtab Farooqui, Melissa C. Skala, David J. Beebe, Jose M. Ayuso

**Affiliations:** 1 Department of Biomedical Engineering, University of Wisconsin-Madison, 1550 Engineering Drive, Madison, WI 53706, USA; virumbralesm@wisc.edu (M.V.-M.); mfarooqui@wisc.edu (M.F.); 2 The University of Wisconsin Carbone Cancer Center, University of Wisconsin, Madison, WI 53705, USA; 3 School of Medicine and Public Health, University of Wisconsin-Madison, 750 Highland Avenue, Madison, WI 53726, USA; 4 Department of Chemistry, University of Wisconsin-Madison, 1101 University Avenue, Madison, WI 53706, USA; mliving43@gmail.com; 5 Morgridge Institute for Research, 330 N Orchard street, Madison, WI 53715, USA

**Keywords:** polystyrene, microfluidics, lumen

## Abstract

Luminal geometries are common structures in biology, which are challenging to mimic using conventional in vitro techniques based on the use of Petri dishes. In this context, microfluidic systems can mimic the lumen geometry, enabling a large variety of studies. However, most microfluidic models still rely on polydimethylsiloxane (PDMS), a material that is not amenable for high-throughput fabrication and presents some limitations compared with other materials such as polystyrene. Thus, we have developed a microfluidic device array to generate multiple bio-relevant luminal structures utilizing polystyrene and micro-milling. This platform offers a scalable alternative to conventional microfluidic devices designed in PDMS. Additionally, the use of polystyrene has well described advantages, such as lower permeability to hydrophobic molecules compared with PDMS, while maintaining excellent viability and optical properties. Breast cancer cells cultured in the devices exhibited high cell viability similar to PDMS-based microdevices. Further, co-culture experiments with different breast cell types showed the potential of the model to study breast cancer invasion. Finally, we demonstrated the potential of the microfluidic array for drug screening, testing chemotherapy drugs and photodynamic therapy agents for breast cancer.

## 1. Introduction

The average number of new drugs approved by the US Food and Drug Administration (FDA) per year has declined since the 1990s, while costs have steadily increased over the years [1]. Specifically, anti-cancer drugs show some of the highest attrition rates among all the therapeutic areas [2]. Many authors argue that current in vitro models fail to mimic relevant cues in the microenvironment, which in turn impacts the results of drug-testing efficiency [3]. Lately, the field has emphasized the need for improving traditional drug testing platforms to provide an increased correlation to clinical success [4]. To address these concerns, researchers have speculated that a more physiologically relevant in vitro culture environment would encourage cells to behave more similarly to cells grown in vivo, therefore increasing the predictability of in vitro models [5].

Breast cancer is the most common cancer in women, affecting one in eight women during their lifetime [6]. Breast cancer originates in the mammary duct, and eventually tumor cells break the wall of the lumen and invade the surrounding tissue. Despite their common origin, breast tumors are highly heterogeneous from both a histopathological (e.g., ductal carcinoma in situ, invasive ductal carcinoma) and molecular (e.g., HER-2^+^, PR^+^, ER^+^) perspective. Additionally, microenvironment alterations including hypoxia, presence of stromal cells critically influence cancer development, and progression are highlighted in breast cancer [7,8,9,10]. The vast number of parameters involved in breast cancer evolution requires new research tools that control costs yet allow for multiple replicates of microenvironmental conditions. In vitro models are well suited for this high-throughput analysis, but polystyrene-based Petri dishes that are traditionally used for in vitro studies do not reliably capture tissue complexity [11]. In this context, microfluidic-based models offer great potential to mimic the tissue architecture. More specifically, the use of microfluidic platforms to generate breast ductal structures can help reproduce the breast cancer microenvironment [12,13]. Using such platforms, we have previously studied the metabolic adaptations undergone by tumor cells inside of the mammary duct; the effect of fibroblasts in breast cancer drug response; and the impact of obesity and adipocytes in hormonal therapy [14,15,16]. However, the platform described in these studies, as well as most microdevices developed by other groups, are based on polydimethylsiloxane (PDMS) [17]. Although PDMS has excellent optical clarity, flexibility, and gas permeation properties, PDMS can nonspecifically absorb small molecules, including certain drugs [17]. Polystyrene (PS) presents a more promising alternative due to its mass-production potential, impermeability to small hydrophobic molecules, and biocompatibility [18].

In this work we have developed an array of microdevices in polystyrene using micro-milling techniques for fabrication. This microfluidic array allows the generation of multiple luminal structures embedded in a 3D extracellular matrix for high-throughput screening. We demonstrated a lower diffusion of hydrophobic molecules into the PS device as compared to a similar comparable PDMS-based microdevice. As a proof of concept, we filled our device with collagen I and lined our resulting luminal structures with breast cancer cells within each device in the array. We monitored breast cancer invasion in the array by using co-cultures of cell lines with varying invasion potential. Additionally, we tested the model for use drug screening exposing the in vitro lumen models to the chemotherapy agent doxorubicin. Finally, we performed photodynamic therapy (PDT) in the lumen. PDT is based on the use of compounds that become toxic after light exposure (i.e., photosensitizers), allowing for a more localized delivery of active compounds, selectively targeting the tumor, and minimizing side effects in the normal tissue and other organs.

## 2. Results and Discussion

### 2.1. Microarray Fabrication and Operation

**Figure 1:**
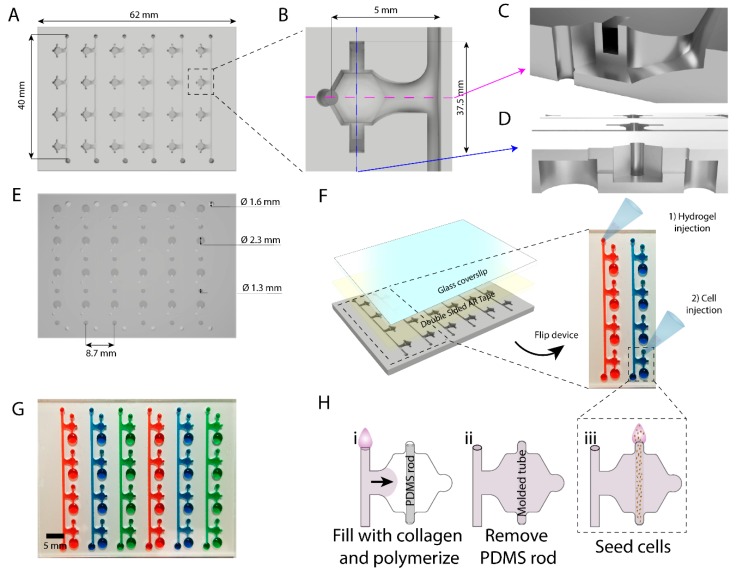
Design and operation of the microfluidic array. (**A**) Top-down schematic view of the microfluidic array. (**B**) Top-down view of one of the devices. (**C**) Inset and magnification of one microdevice in the microfluidic array with marked cross-sections in (**D**) (pink cross-section) and (**E**) (blue cross-section). (**F**) The microfluidic array is milled on both sides of the polystyrene (PS) piece, and a glass coverslip is used to seal the lumen side with cell culture-grade double sided Advanced Research tape. (**G**) Each line of devices is sealed from adjacent lines, allowing for testing of multiple doses in quadruplet of target compound in one microdevice array. (**H**) Schematic of collagen filling and cell seeding in each of the devices in the microfluidic array.

The microfluidic array was based on an original design described previously by us [13]. However, this design was fabricated in PDMS via soft lithography. To improve on some of the design drawbacks of that device (e.g., labor-intense fabrication and design operation, PDMS sequestration of small hydrophobic molecules), we designed a PS-based version of the device [18]. Firstly, the device was designed to include multiple micro-culture chambers matching the spacing distance center-to-center used in conventional 96 well-plates for compatibility with multi-channel micro-pipettes, automated pipetting systems, and automated imaging platforms (i.e., plate readers) (Figure 1A–E). This feature diminished user-to-device interaction and made the platform more amenable to current high throughput screening tools. Next, the design included a side microchannel that connected several devices together. The dimensions of the hydrogel loading microchannel ensured fluid with different viscosities (e.g., water, PBS, collagen, Matrigel) spontaneously flowed via capillary forces through the microchannel and filled the microchambers [18,19]. Once the hydrogel is polymerized, the sacrificial PDMS rod can be removed for cell seeding as required (Figure 1H), therefore it does not get in contact with cells or cell culture media. Thereafter, the design leverages passive pumping to spontaneously perfuse the tubular space left by the rod, facilitating cell seeding, and flow of different solutions (e.g., media, drug solutions) [13].

Another novelty in the design is that milling patterns were gradually equilibrated with a transition in height difference between the loading channel and device ceiling to minimize capillary valve occurrence [20]. This enabled pre-polymerized hydrogels to wick across the surface into the body of the device allowing for quick hydrogel loading. Double sided milling was used for this device, to achieve lumen and port overlap in the design. Once milled, cell culture grade double-sided tape was used to attach a glass coverslip to the bottom, enclosing the microdevice lumen chamber, and leaving lumen and loading ports open on the opposite side of the polystyrene platform. The double-sided tape used has extensively been used for cell culture, and therefore its biocompatibility has been demonstrated before [21]. To ensure appropriate isolation between different lumen arrays, we perfused water solutions of different food coloring, and observed no mixing of colors or colored water between the chambers overtime (i.e., 24 h) (Figure 1G). This successful compartmentalization allows for testing of different culture conditions in each line of connected devices, and therefore makes this platform amenable to higher throughput screening.

### 2.2. Drug Diffusion in the Microarray

**Figure 2:**
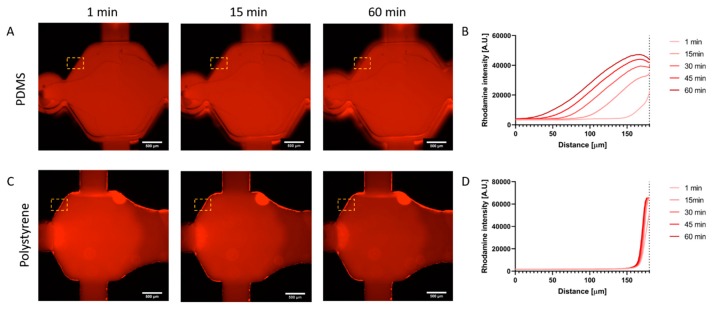
Diffusion profile. (**A**) A Rhodamine B solution (in red) was injected into polydimethylsiloxane (PDMS)-based microdevices, and Rhodamine B diffusion into the material was monitored with real-time microscopy. Images showed Rhodamine B penetrated into the PDMS bulk material. (**B**) Graph shows the Rhodamine B diffusion profile. Rhodamine B fluorescence was analyzed across the delimited region (yellow rectangle). The location of the microdevice wall is denoted with dashed line. (**C**) Rhodamine diffusion experiment in the PS-based microfluidic array. No Rhodamine B diffused or leaked into the material. (**D**) Rhodamine B diffusion profile in the microfluidic array.

Microfluidic devices for high-throughput applications such as drug screening must ensure liquids are completely confined in the internal structures of the microdevice, preventing any potential leakage. Additionally, PDMS has been reported to be permeable to small hydrophobic molecules. Thus, we decided to compare molecule absorption in the PDMS-based microdevices and the PS microfluidic array. In this context, a Rhodamine B solution was perfused in the central microchamber of both microdevices (i.e., PS- and PDMS-based microdevices). Rhodamine B is a hydrophobic compound that naturally fluoresces in red, which makes monitoring with real-time microscope possible. Minutes after injection, Rhodamine B penetrated the PDMS of the PDMS-based microdevice, leading to an observable fluorescence front (Figure 2A) with the 50% maximum intensity located at 120 µm into the PDMS. Conversely, the PS-based microfluidic array showed (Figure 2C,D) showed no Rhodamine B penetration into the PS or leakage between the different layers forming the microarray. This suggests that small molecules and lipophilic compounds would be more retained in PDMS-based devices as opposed to PS-based microfluidic arrays. These results also suggest hydrophobic absorption in the PDMS-based device, but not in the PS-based microfluidic array. The inertness of cell culture platforms is crucial for long-term cell culture viability, normal cell function and metabolism, and drug testing applications. Therefore, we anticipate that this device could solve reported problems of drug retention in microfluidic drug testing assays.

### 2.3. Cell Invasion

**Figure 3:**
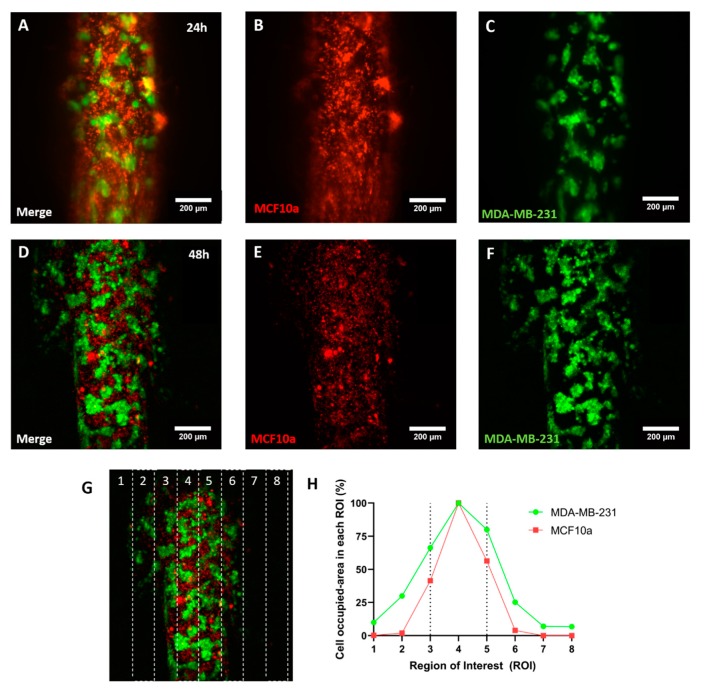
Cell migration. (**A**–**C**) confocal images showing MCD10A cells labelled with vibrant Dil (in red) and MDA-MB-231 GFP cells at 3:1 ratio after 24 h in culture. The confocal images showed no observable cell migration. (**D**–**F**) Confocal images after 48 h showing MCF10A and MDA-MB-231 invading the hydrogel. (**G**,**H**) Migration analysis. The confocal image was vertically divided in 8 columns and the area occupied by MCF10A (in red) or MDA-MB-231 GFP (in green) was analyzed, normalized, and plotted.

Breast cancer originates in the mammary duct and if left untreated, ultimately invades into the surrounding tissue. Multiple studies have shown that the molecular mechanisms involved in breast tumor invasion offer potential targets for new cancer therapies. Therefore, we evaluated the potential of the microfluidic array to monitor breast cancer invasion. To assess invasion potential, we mixed two different cell lines labelled with different fluorophores to monitor cell migration into the surrounding matrix. MCF10A cells, a model of normal mammary epithelial cell, were labelled in red with the fluorescent lipid vibrant Dil. MDA-MB-231 transduced with green fluorescent protein (GFP) were used as a model of highly invasive breast cancer cell [22]. MCF10A and MDA-MB-231 were mixed at 3:1 ratio and injected into the lumen (Z-stack of luminal structure is shown in Supplementary Video S1). After 24 h in culture, the cells remained confined in the lumen, mimicking behavior of these cell types in previous lumen-based studies (Figure 3A–C) [12,14]. However, after 48 h, migration was observed in both cell types (Figure 3D–F). Interestingly, the results demonstrated both cell lines invaded the surrounding tissue, with MDA-MB-231 located at the invading edge of this migration process (Figure 3G,H, ROI 1-2 and 6-7). Previous papers have shown that epithelial cells, including normal breast and cancer cells, can exhibit collective migration, where a few leader cells lead the invasion with a majority of follower cells behind [23]. The molecular machinery used in collective migration is unique and different from other migration mechanisms (e.g., mesenchymal, ameboid migration), which highlights the potential of in vitro platforms to identify collective migration inhibitors. Further, in the case of this microfluidic array, the design decreases the need for user handling while enabling multiple condition screening and monitoring.

### 2.4. Drug Screening

**Figure 4:**
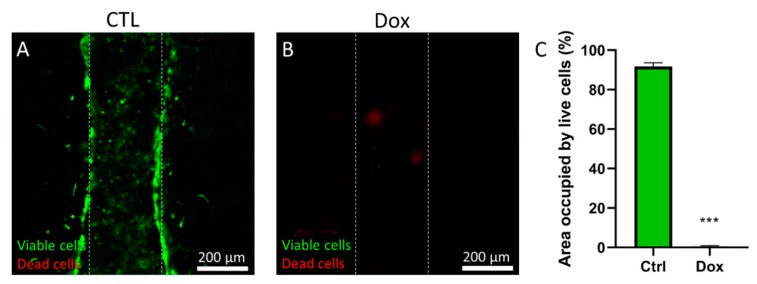
Effect of Doxorubicin on cell viability. (**A**) GFP^+^ MDA-MB-231 cells were cultured in the lumen. After 72 h in culture, a solution containing propidium iodide was perfused through the lumen to stain dead cells in red and cell viability was observed by confocal microscopy. GFP^+^ MDA-MB-231 cells showed high viability in the absence of doxorubicin. (**B**) GFP^+^ MDA-MB-231 cells were cultured for 3 days in 100 µM doxorubicin. Cell viability images showed a significant decrease in cell viability and most dead cells were removed from the lumen during the media changes and the staining process. *** *p* < 0.001.

The development of new chemotherapy agents still relies on in vitro platforms during the preclinical phase of drug development [24]. Thus, we decided to explore the capacity of the model as new in vitro tool for drug screening. For this proof of concept, we chose to use doxorubicin, a classical treatment for breast cancer considered one of the most potent chemotherapeutics for this disease [25]. GFP^+^ MDA-MB-231 cells were seeded in the lumen at 5 million cells/mL following the protocols described above. After 24 h in culture, the chemotherapy agent doxorubicin was perfused through the lumen at 100 µM and the microfluidic array was placed in the incubator. After 3 days in culture, a solution containing propidium iodide (PI) was perfused through the lumen to label dead cells in red, and cell viability was analyzed by confocal microscopy (Figure 4). The results showed that in control conditions (Figure 4A) GFP^+^ MDA-MB-231 cells covered most of the lumen area (91.7%, Figure 4C) whereas the addition of doxorubicin led to a significant reduction in cell viability (Figure 4B,C). These results illustrate some of the potential drug testing readouts expected for our platform. Likewise, our results corroborate the adequacy of the PS-based microfluidic arrays for drug development and testing applications.

### 2.5. Photodynamic Therapy (PDT)

**Figure 5:**
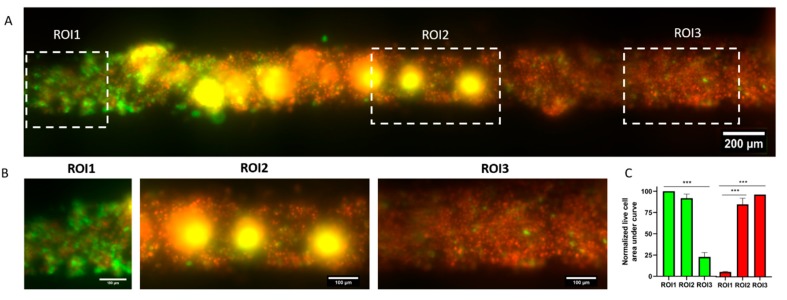
Photodynamic therapy in the microfluidic array. (**A**) MDA-MB-231 green fluorescent protein (GFP) cells were injected in the lumen and incubated with 500 ng/mL verteporfin for 24 h. The right end of the lumen was exposed to 485/35 nm light for 45 s to photoactivate verteporfin. After another 24 h, cell viability was evaluated, showing a gradient of viability across the lumen. (**B**) Images showing the left, center, and right section of the lumen. (**C**) Graphs showing the normalized area under the curve of the luminescence plot for live cell (green) and dead cell (fluorescence) in the left, central, and right region of the lumen. *** *p* ≤ 0.001.

Photodynamic therapy (PDT) is a treatment that uses a drug, called a photosensitizer or photosensitizing agent. Upon exposure of photosensitizers to a specific wavelength of light, they produce reactive oxygen species that kill nearby cells [26,27]. Some PDT photosensitizers have FDA approval to treat metastatic breast cancer [28]. To explore PDT in the system, GFP^+^ MDA-MB-231 cells were cultured in the lumen for 24 h and then the photosensitizer verteporfin was added at 500 ng/mL. Then, the right end of the lumen was exposed to 485 nm light for 45 s using a 10× objective to activate the photosensitizer. The microfluidic array was placed in the incubator again and cell viability was analyzed 24 h later. The images revealed a gradient of cell viability across the lumen (Figure 5A), exhibiting high cell viability in the left side of the lumen (Figure 5B,C) that decreased at the center of the lumen and reached the minimum in the right side of the lumen (i.e., the exposed area). The use of the PS-based microfluidic array provides with a long 3D tubular structure that enables drug testing for new therapeutic options like PDT. This platform enabled PDT tests that resolved the extent of the cytotoxic capabilities of PDT therapy in a mammary duct-mimicking tubular struture. The use of this microdevice could provide insight into the conditions needed to activate PDT and its expected spatial and cytotoxic impact. These studies would be beneficial for both basic science and precision oncology approaches.

## 3. Methods

### 3.1. Cell Culture

MCF10A human mammary epithelial cells were obtained from ATCC (ATCC^®^CRL-10317™). MCF7 human mammary epithelial cells from metastatic site were obtained for ATCC as well (ATCC^®^ HTB-22™) Human mammary adenocarcinoma cells, MDA-MB-231, both wild type and transfected to stably expressing green fluorescent protein (GFP), were a kind gift from Dr. Suzanne Ponik (University of Wisconsin, Madison, WI 53705, USA). All cells were maintained with RPMI base media (Gibco, 11875) with 10% fetal bovine serum (FBS, serum (VWR, 97068-085) and 1% penicillin/streptomycin (ThermoFisher, 15140-122, Grand Island, NY, USA) on cell culture-treated flasks (Corning, 156499, Oneonta, NY, USA). Supplemented RPMI media is referred to as relevant media in the rest of the paper. Cells were harvested via standard trypsinization. Briefly, cells were washed with PBS 1× (diluted from 10× with distilled water, Thermo Fisher BP3991), incubated with a 0.25% trypsin/EDTA solution (LifeTechnologies, 25200056, Fitchburg, WI, USA) for 5 min. Trypsin was inactivated with relevant media. Next, cells were pelleted at 300 g for 4 min and finally resuspended to the desired concentration for subsequent experiments.

### 3.2. PDMS Device Fabrication

Standard SU-8 photolithography was used to create the device architecture as previously described [13]. PDMS was mixed at a 10:1 polymer:curing agent ratio, poured onto SU-8 masters and used to fill 23G needles (BD, 305145). PDMS mixture was baked for 4 h at 80 °C. After baking, PDMS was extracted from the needles to form sacrificial PDMS rods. PDMS layers were detached from the SU-8 masters, ethanol bonded together with the sacrificial PDMS rod in place. Assembled devices were then plasma bonded to a 0.9 mm thick glass coverslip. To ensure collagen adhesion to PDMS surface, the devices were treated with a 0.2% aqueous poly(ethyleneimine) (PEI, Millipore-Sigma, 408727, St Louis, MO, USA) solution for 10 min, then with a 0.04% aqueous glutaraldehyde (GA, Millipore-Sigma, 354400, St Louis, MO, USA) solution for 30 min, then aspirated. To rinse residual PEI and GA, the devices were washed with deionized water three times.

### 3.3. Polystyrene Device Fabrication

Devices were micro-milled on a CNC mill out of sheets of 2 mm polystyrene (PS) with a 0.02 inch end-mill using a two-sided milling technique. We milled the hydrogel filling channel, the connection channels and all the devices into one side of the 2 mm thick PS sheet. Then, the platform was flipped upside-down and milled on the other surface. On this side of the PS, the ports for the devices and the filling channels were milled. Cell seeding ports (small and large) were milled at 1.5 mm-depth. The cell seeding ports and body of the device vertically overlapped by 0.5 mm, making room for the sacrificial PDMS rod to be suspended and therefore not in contact with the bottom surface of the platform. Milled devices were rinsed thoroughly with deionized water and dried with a high-pressure air stream. Sacrificial PDMS rods were loaded into individual lumen devices using fine tweezers. The flexibility of PDMS is required for the functionality of the rods, and therefore their fabrication with PS was not considered. The back of the PS devices was exposed to oxygen plasma to render the surface hydrophilic. Double-sided AR tape (AR care8890) was cut to the size of the PS device and adhered to a coverslip (50 × 75 mm, Corning 2947-75X50) to allow for a complete seal and optical transparency. Devices were flipped in order to access loading ports for further use.

### 3.4. Rhodamine B Diffusion

Rhodamine B (Millipore-Sigma, 83689, St Louis, MO, USA) was dissolved at 5 mg/mL in distilled water, and later diluted 1:100 in PBS. PDMS and PS fabricated devices were both filled with the Rhodamine B solution. Rhodamine B fluorescence was tracked via fluorescent microscopy for 1 h. The solution was then incubated at room temperature for 48 h and imaged again. Finally, the Rhodamine B solution was aspirated, and devices were washed thrice for 20 min each time before a final fluorescence image acquisition. ImageJ software was used to generate fluorescence plot profiles in the regions of interest (ROI) located at the edge of the chamber, and into the material.

### 3.5. Collagen Loading in Devices

200 µl of Rat Tail Collagen I, High Concentration (Corning, 354249, Bedford, MA, USA, stock at 8.28 mg/mL) was neutralized with 5 µl of 0.5 M NaOH (Sigma, S5881, St Louis, MO, USA) and diluted to 4.5 mg/mL. Relevant osmolarity was achieved using 40 µl PBS10× (Fisher Scientific, BP3991, Fair Lawn, NJ, 07410, USA) with 155 µL of sterile distilled water. The mixture was kept on ice for 20 min prior to loading the devices. pH was measured using pH Test Strips (Whatman, 2629-990, Little Chalfont, UK), and only mixtures with pH values of 7–7.4 were used for experiments. PS devices were thoroughly rinsed with deionized water twice and dried using vacuum aspiration. During collagen loading, the devices were placed on a metal block on top of ice to prevent the collagen solution from polymerizing until the devices were fully filled. Approximately 140 µL of collagen solution were injected in each line port to fill the connected line of lumen devices, whereas 10 µL were used per PDMS lumen device. Once all devices were filled, they were left to polymerize at room temperature for 10 min. After that, the devices were transferred to an omni-tray plate (Thermo, 242811, Rochester, NY, USA) and incubated at 37 °C for 30 min prior to cell loading.

### 3.6. Vybrant Staining

MCF10a cells were stained in red with the fluorescent lipid Dil (Thermo Fisher, V22889, Eugene, OR, USA) according to manufacturer’s instructions. Briefly, MFC10a cells were resuspended at 5 cells million/mL in PBS. DiI was added at a 1:200 dilution from the stock and incubated at 37 °C protected from light for 10 min. Next, cells were pelleted and washed twice with PBS before resuspending in relevant media at the desired concentration (20 million cells/mL).

### 3.7. Cell Loading into Lumen Devices

Sacrificial PDMS rods were removed from each device and the resulting hollow lumen was filled with relevant media. Cells were resuspended at the desired concentration of 6 million/mL. Three microliters of the cell solution were pipetted into the small port connected to the luminal geometry and passively pumped through the lumen. Cultures were incubated at 37 °C for 15 min at a time and flipped 3 times (a total of 1 h of incubation) so that the cells adhered to the entire surface of luminal geometry. After, excess media was gently aspirated to remove unattached cells. Devices were cultured overnight at 37 °C, and media was replenished immediately the following morning. Experimental conditions were introduced 24 h after seeding, replenishing every 24 h.

### 3.8. Cell Viability

Calcein AM (Thermo Fisher, C1430, Eugene, OR, USA) and propidium iodide (Millipore Sigma, P4170, St Louis, MO, USA) were resuspended in DMSO at 5 mM and 2 mg/mL respectively, and then diluted in PBS to final concentrations of 5 µM and 2 µg/mL respectively. The cells were stained in this solution for 30 min at 37 °C prior to confocal imaging with an SP8 Leica microscope with stimulated emission depletion microscopy (STED) module. Green fluorescence was detected using an excitation laser 485 nm, emission filter 495–525 nm. Red fluorescence was detected using an excitation laser, 532 nm, 540–600 nm. The number of propidium iodide positive (dead, red) cells were counted and divided by the total number of cells (calcein AM positive, green; and PI positive cells, red) to calculate the percentage of viable cells.

### 3.9. Invasion Assay

MCF10a cells were stained with DiI as described in previous sections and resuspended at 20 million cells/mL. GFP-fluorescent MDA-MB-231 cells were resuspended at 6 million/mL. Both cell suspensions were mixed 1:1 and loaded through the lumens in PS devices prepared as previously described. Devices were imaged with a confocal microscope (Leica SP8 with STED module) at 24 and 48 h. Cell invasion was quantified as the area occupied by cells in different ROIs defined in the captured field with ImageJ, both for green and red fluorescence.

### 3.10. Photodynamic Therapy Experiment

PS lumen devices were prepared as described above to create tubular void structures embedded in hydrogel. GFP fluorescent MDA-MB-231 cells were resuspended at 5 million cells/mL and loaded into the lumen structure as described above and left to adhere for 24 h. Verteporfin (Selleckchem, S1786, Pittsburgh, PA, USA), was dissolved at 25 mg/mL in DMSO, and later diluted 1:50,000 (to a final concentration of 500 ng/mL) in relevant media. Verteporfin solution was perfused through the lumen and incubated at 37 °C for 1 h. Thereafter, cells in the lumen were exposed to fluorescent light using a Nikon Eclipse Ti microscope and a 485/35 nm filter for 45 s. After the light activation of verteporfin, cells were left in the incubator overnight. Viability was assessed via propidium iodide staining as described in subsequent sections, since cells were already fluorescently green, calcein was not required to stain live cells. Images were analyzed using ImageJ by plotting the fluorescence profile in three different region of interests (ROIs) in the image within the lumen (at the beginning, center and end of the lumen along the axis). The area under the curve was calculated and normalized to the highest value (among green and red) to demonstrate which of the fluorescence channels was more prevalent in each section (and ROI) of the lumen.

### 3.11. Doxorubicin Cytotoxicity Assay

Doxorubicin was dissolved in DMSO at 100 mM, and then dissolved 1:1000 in culture media. PS microarrays were prepared as previously described and MDA-MB-231 were loaded in the lumen at 5 million cells/mL. Cells were left to adhere for 24 h and then the doxorubicin solution was added and incubated for 72 h. Then, viability was measured using calcein AM and propidium iodide as described in the following section.

### 3.12. Statistical Analysis

All the experiments were repeated at least three times as independent biological replicates. All results are presented as the mean ± standard deviation. Data were analyzed using GraphPad Prism v7 and statistical significance was set at *p* < 0.05. First, normal distribution was assessed by the Shapiro-Wilk test. If the normality test was passed, a Student’s T-test was performed. Multiple comparisons by ANOVA were corrected using Tukey’s tests.

## 4. Conclusions

The pharmaceutical industry now relies on high throughput systems to test thousands of compounds a year. However, most used systems have shown to produce confounding results that do not fully recapitulate in vivo effects of drugs. On the other hand, microfluidics can provide very useful models that recapitulate functions of in vivo systems more accurately, therefore providing with great opportunities for more relevant in vitro drug testing. However, most microfluidic devices rely on PDMS, which have important drawbacks for drug testing. Among these drawbacks, the use of highly hydrophobic materials, which could impact the effective concentration of molecules (e.g., drugs) in the cell culture system; and the labor-intensive protocols are important limitations for potential users to adopt microfluidic drug testing. In an effort to increase the throughput of microfluidic organotypic models of breast cancer, as well as decrease user handling, we have developed a lumen array platform made of polystyrene. We have leveraged microfluidic physical principles (i.e., capillarity, passive pumping), to decrease user operation compared to traditional PDMS-based microfluidic devices and increase throughput by incorporating a side channel to fill several microdevices with hydrogel solution at the same time. Compared to PDMS, PS has low retention capabilities of lipophilic molecules and is widely accepted among in vitro-focused researchers. We have illustrated this premise by tracking Rhodamine B diffusion in our microfluidic lumen array, revealing minimal Rhodamine B penetration in the PS-based device. Likewise, we have demonstrated three cell-based applications for breast cancer in this platform. First, we have assessed cancer cell invasion in a model of both normal (MCF10a) and invasive breast cancer cells (MDA-MB-231) and demonstrated a higher invasiveness of MDA-MB-231 cells in the surrounding matrix. Next, we have used doxorubicin, a common chemotherapeutic, and assessed its cytotoxicity in a lumen model of breast cancer cells. Finally, we have leveraged our model to test the cytotoxicity and reach of photodynamic therapy (PDT) with verteporfin, showing a gradient of cell cytotoxicity along the lumen.

## Supplementary Materials

The following are available online, Figure S1: Fluorescence microscopy image of MCF10a (red) and MDA-MB-231 transfected with green fluorescent protein (green), immediately after adhesion. Video S1: Z-stack video of MCF10a (red) and MDA-MB-231 transfected with green fluorescent protein (green), immediately after adhesion.
