# Supplementary material for: Development of a Microfluidic Array to Study Drug Response in Breast Cancer
Source: Molecules. 2019 Nov 30;24(23):4385. doi: 10.3390/molecules24234385 (PMC6930663; doi:10.3390/molecules24234385)
Supplement: Supplementary file 1 [file molecules-24-04385-s001.zip › molecules-646545 supplementary.pdf]

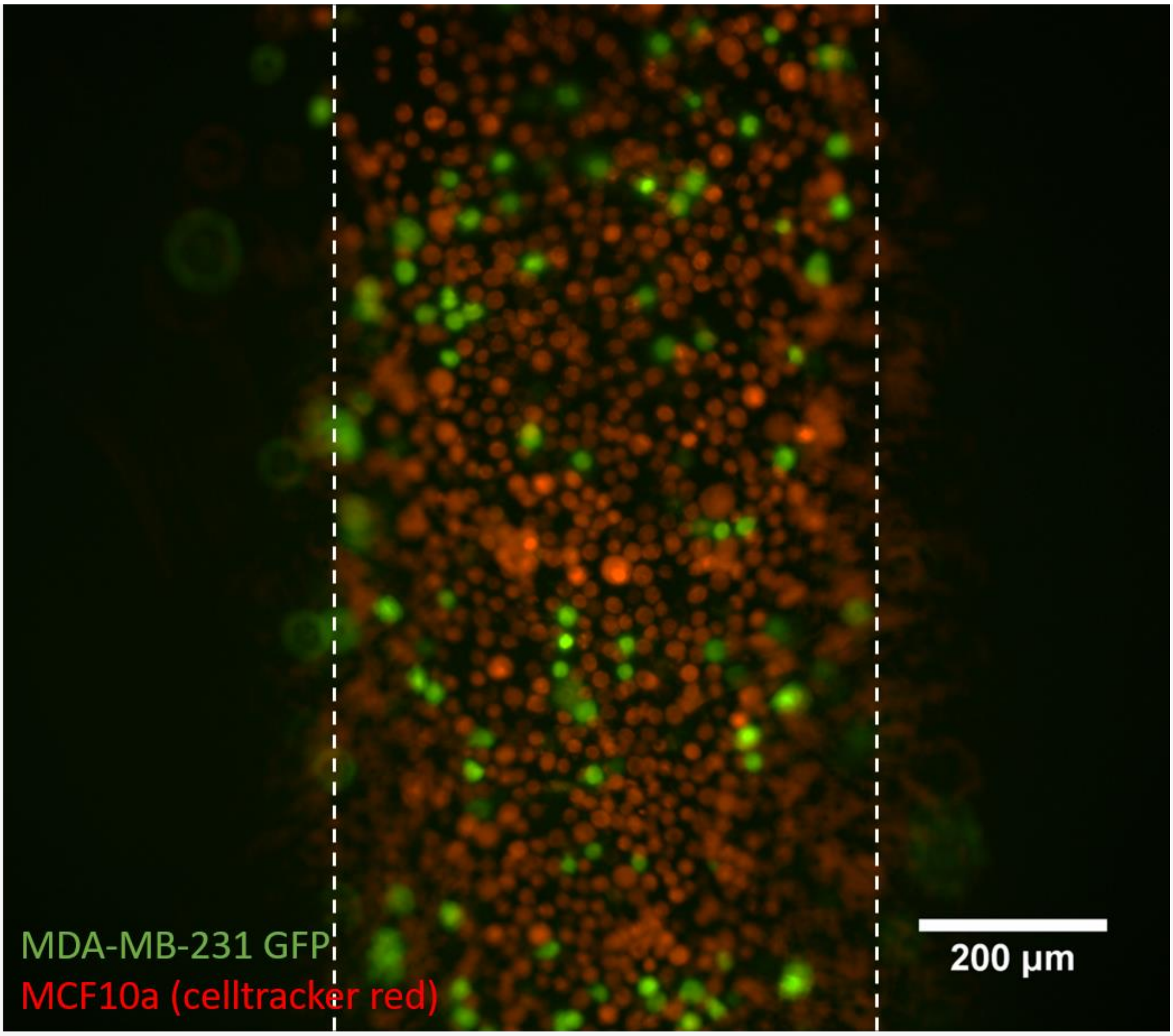

Figure 1. Fluorescence microscopy image of MCF10a (red) and MDA-MB-231 transfected with green fluorescent protein (green), immediately after adhesion.
